# Transcriptional Analysis of Human Skin Lesions Identifies Tryptophan-2,3-Deoxygenase as a Restriction Factor for Cutaneous *Leishmania*

**DOI:** 10.3389/fcimb.2019.00338

**Published:** 2019-10-04

**Authors:** Vasco Rodrigues, Sónia André, Hasnaa Maksouri, Tarik Mouttaki, Soumiya Chiheb, Myriam Riyad, Khadija Akarid, Jérôme Estaquier

**Affiliations:** ^1^CNRS-ERL3649, Université Paris Descartes, Paris, France; ^2^Research Team on Immunopathology of Infectious and Systemic Diseases, Laboratory of Cellular and Molecular Pathology, Faculty of Medicine and Pharmacy, Hassan II University of Casablanca, Casablanca, Morocco; ^3^Department of Dermatology, University Hospital Ibn Rochd of Casablanca, Casablanca, Morocco; ^4^Laboratory of Parasitology, Faculty of Medicine and Pharmacy, University of Hassan II Casablanca (UH2C), Casablanca, Morocco; ^5^Molecular Genetics and Immunophysiopathology Research Team, Health and Environment Laboratory, Aïn Chock Faculty of Sciences, University of Hassan II Casablanca (UH2C), Casablanca, Morocco; ^6^Faculty of Medicine, Centre Hospitalier Universitaire (CHU) de Québec Research Center, Laval University, Quebec, QC, Canada

**Keywords:** cutaneous leishmanaisis, tryptophan-2, 3-dioxygenase, *Leishmania tropica*, *Leishmania major*, parasite load

## Abstract

Disease manifestation after infection with cutaneous *Leishmania* species is the result of a complex interplay of diverse factors, including the immune status of the host, the infecting parasite species, or the parasite load at the lesion site. Understanding how these factors impact on the pathology of cutaneous leishmaniasis (CL) may provide new targets to manage the infection and improve clinical outcome. We quantified the relative expression of 170 genes involved in a diverse range of biological processes, in the skin biopsies from patients afflicted with CL caused by infection with either *L. major* or *L. tropica*. As compared to healthy skin, CL lesions bear elevated levels of transcripts involved in the immune response, and conversely, present a significant downregulation in the expression of genes involved in epidermal integrity and arginine or fatty acid metabolism. The expression of transcripts encoding for cytotoxic mediators and chemokines in lesions was inversely correlated with the expression of genes involved in epidermal integrity, suggesting that cytotoxicity is a major mediator of CL pathology. When comparing the transcriptional profiles of lesions caused by either *L. major* or *L. tropica*, we found them to be very similar, the later presenting an aggravated inflammatory/cytotoxic profile. Finally, we identified genes positively correlated with the parasite load in lesions. Among others, these included Th2 or regulatory cytokines, such as *IL4* or *IL10*. Remarkably, a single gene among our dataset, encoding for tryptophan-2,3-deoxygenase (TDO), presented a negative correlation with the parasite load, suggesting that its expression may restrict parasite numbers in lesions. In agreement, treatment of macrophages infected with *L. major in vitro* with a TDO inhibitor led to an increase in parasite transcripts. Our work provides new insights into the factors that impact CL pathology and identifies TDO as a restriction factor for cutaneous *Leishmania*.

## Introduction

Protozoan parasites of the genus *Leishmania* cause a group of diseases collectively known as leishmaniasis, with a clinical spectrum ranging from localized skin lesions to systemic visceral disease. *Leishmania* shuttles between an insect vector and a mammalian host to successfully complete its life cycle. In the gut of female sandflies, *Leishmania* promastigotes go through a maturation process that gives rise to infectious metacyclic forms, which are transmitted to a mammal's skin during blood feeding. These inoculated promastigotes are rapidly taken up by resident or recruited phagocytes and differentiate into the intracellular amastigote stage that spreads the infection (Kaye and Scott, 2011).

The cutaneous form of leishmaniasis remains a public health problem across many regions of the globe, with an estimated global incidence of 1 million cases (David and Craft, 2009; Alvar et al., 2012). In the Old World, cutaneous leishmaniasis (CL) is usually caused by *L. major, L. tropica*, or *L. aethiopica*, while in the Americas, the infecting species are typically *L. braziliensis* or *L. amazonensis* (Murray et al., 2005; WHO, 2013). In Morocco, CL is caused mainly by *L. major* and *L. tropica* and remains a significant health problem, with a steady increase in the number of reported cases since the beginning of the century, as a result of the emergence of several new foci (Aoun and Bouratbine, 2014; Mniouil et al., 2017).

Cutaneous *Leishmania* infection starts with an asymptomatic period of variable duration, characterized by parasite proliferation. This silent phase ends with the appearance of a small erythema at the site of the bite, indicating the onset of an inflammatory response in the infected tissue. The progressive infiltration of neutrophils, macrophages, eosinophils, or T cells heightens the inflammatory response and leads to the development of a papule or a nodule that grows slowly, over several weeks. A crust appears centrally and eventually falls off, exposing the ulcerated lesion. Ulcers generally present raised and indurated margins, healing gradually over months or years (Murray et al., 2005; Reithinger et al., 2007). Disease manifestation is highly variable, depending on the immune status of the host and the infecting parasite species. For instance, in North Africa, cutaneous lesions caused by *L. major* tend to be exudative or “wet,” large and complicated by superficial and secondary bacterial infections. They are typically multiple and located on limbs. Spontaneous healing but with indelible scars is obtained in <8 months. Lesions caused by *L. tropica* are often “dry” with a central crust, mainly single and located on the face. Some *L. tropica* lesions last more than 1 year, confirming the chronic tendency of this form of CL. Relapses and treatment failures are also not exceptional. Hence, infection caused by *L. tropica* seems to be more insidious compared with *L. major* infection, with a longer incubation period. However, multiple, inflammatory, and infiltrative diffuse lesions were described in some Moroccan outbreaks (Aoun and Bouratbine, 2014).

Over the past decades, countless studies in animal models and human patients helped to elucidate how immune responses are orchestrated during CL and how they translate into the wide spectrum of clinical manifestations observed in CL patients (Scott and Novais, 2016). The crucial role played by Th1 immune responses in the resolution of infection is well-established, as it leads to the production of IFN-γ and TNF that activate macrophages to kill intracellular amastigotes. However, the excessive induction of Th1 responses may lead to immunopathology. Similarly, the presence of CD8 T cells expressing cytotoxic markers, such as perforin and granzymes, in lesional tissue has recently been associated with tissue damage that manifests as skin ulceration (Scott and Novais, 2016; Novais et al., 2017). In contrast, the action of immune-regulatory factors such as IL-10 or TGF-β promotes parasite persistence and lesion chronicity. These cytokines impair the induction of protective responses and may lead to an aggravated form of the disease known as diffuse cutaneous leishmaniasis (DCL), whose hallmark is a depressed cellular immunity as evidenced by a negative delayed type hypersensitivity response (Scott and Novais, 2016).

Studies in human CL patients have classically evaluated the capacity of circulating leukocytes to produce cytokines or proliferate when exposed to parasite antigens (Farajnia et al., 2005; Vargas-Inchaustegui et al., 2010; Shahi et al., 2013). While they may provide good correlates of protection, these systemic responses seldom reflect the immune environment in the infected tissue. More recently, transcriptomic and proteomic approaches provided a more detailed picture of the local immune response at the lesion site (Maretti-Mira et al., 2012; da Silva Santos et al., 2015; Novais et al., 2015; Christensen et al., 2016). Nevertheless, studies comparing the transcriptional signatures of CL lesions caused by different *Leishmania* species are still lacking. Also, the parasite load appears to impact the inflammatory and general transcriptional signatures of the lesions (Christensen et al., 2016). However, such knowledge has not yet been explored to identify factors that may promote or restrict parasite growth.

We quantified the transcript abundance of genes involved in inflammation, immunity, metabolism, and epidermal integrity from the skin of human CL patients infected with either *L. major* or *L. tropica*, recovered from Moroccan endemic areas. Confirming previous observations, we found that lesions are transcriptionally distinct from healthy skin, exhibiting a strong co-induction in the transcript levels of immune mediators, genes related with Th1 immune responses or metabolic enzymes. Conversely, genes associated with epidermal integrity and Th2 immune responses were either downregulated or remained unchanged. *L. major* and *L. tropica* lesions had very similar transcriptional signatures, the latter exhibiting an aggravated pro-inflammatory profile. Employing differential gene expression analysis, we also identified several functionally-related genes whose expression was associated with lesions bearing a high parasite load. These included immunoregulatory genes, and genes related with the metabolic function. Conversely, the expression of the tryptophan-catabolizing enzyme tryptophan-2,3-dioxigenase (TDO) correlated negatively with parasite transcripts. Importantly, treating cultured macrophages with a specific TDO inhibitor augmented *L. major* transcripts. Altogether, our results reveal novel gene signatures associated with CL and identify TDO as a restriction factor for cutaneous *Leishmania*.

## Materials and Methods

### Ethics Statement

This work was conducted according to the principles specified in the Declaration of Helsinki and under the local ethical guidelines of the Ethics Committee for Biomedical Research (Faculty of Medicine and Pharmacy, Hassan II University of Casablanca, Morocco) that approved this research. The team explained to the patients the objectives of the survey, and why it needed a cutaneous biopsy, which is used routinely at the Department of Dermatology (University Hospital Ibn Rochd, Casablanca) for the parasitological confirmation of cutaneous leishmaniasis diagnosis before any treatment prescription. The dermatologist asked for the patients' consent (for adults) or from the parents for children. The sampling was done only if the patients or their tutors gave their oral consent. At the time of biopsy sampling (between 2012 and 2014), oral consent was the sole requirement imposed by the Ethics Committee to allow for patient tissue sampling for research purposes and thus written consent was not obtained. Oral consent was also obtained from the healthy skin donors. Finally, the team guaranteed the confidentiality of their personal and clinical data and that the results would be processed anonymously.

Peripheral blood was obtained from healthy adult donors (Etablissement Français du sang, Paris, France). All donors signed informed consent allowing the use of their blood for research purposes.

### Study Subjects, Dermal Samples, Diagnostic, and Species Typing

Nineteen (19) patients presenting cutaneous lesions suggestive of CL were received and sampled at the Department of Dermatology -Ibn Rochd Hospital, Casablanca. Before the beginning of the treatment course, a skin biopsy was collected under sterile conditions from the border of active skin lesions. Normal skin samples were taken from three volunteers that were living in non endemic areas and without a history of cutaneous leishmaniasis. Sampled biopsies were immediately conserved in RNA Later (QIAGEN) at −80°C until RNA extraction.

Diagnosis of cutaneous leishmaniasis was confirmed by direct examination of amastigotes in Giemsa-stained smears of syringe-sucked dermal fluid, by parasite culture in NNN medium from recovered dermal fluid, or by PCR amplification of the kDNA minicircle region using the primer pair 13A/13B, as described (Reale et al., 1999).

*Leishmania* species typing in the lesions was carried out using the Internal Transcriber Sequence-1 (ITS1) PCR-RFLP assay, as previously described (Mouttaki et al., 2014).

### RNA Extraction and Quantitative PCR

Skin biopsies, genotyped as *L. major* or *L. tropica*, or healthy skin controls, were grinded into a fine powder in liquid nitrogen using a mortar and pestle. One mL of TRIzol reagent (Thermo Fisher Scientific) was added to the powder, homogenized, and spun to remove particulate matter. Two hundred microliters of chloroform were added to TRIzol and the aqueous phase recovered after centrifugation. RNA was precipitated with one volume of isopropanol, washed two times in ethanol (70% v/v), and solubilized in H_2_O. The concentration, purity and integrity of the extracted RNAs were verified in a Nanodrop 2000 spectrophotometer and the Experion Automated Electrophoresis System (Bio-Rad).

RNA was reverse transcribed using the AffinityScript QPCR cDNA synthesis kit (Stratagene), following the manufacturer's instructions. Quantitative PCR was performed in 10 μL reactions using the Sybr Green technology and the 7900HT Fast Real-Time PCR System (Applied Biosystems). The thermal cycle consisted of a hold of 15 min at 95°C, followed by 40 cycles of denaturation (95°C, 15 s), annealing (60°C, 30 s) and extension (72°C, 30 s). Primers for host genes used in this study were designed using the AutoPrime software, and their sequence is provided in Table S1. To quantify the relative parasite load in patient biopsies, we employed primers against the *Leishmania* genus-specific genes *KMP11* and *RRNA45*, whose transcript abundance is constant during the parasite life cycle, and have been previously employed as reference genes for quantitative PCR studies in *Leishmania* (Moreira et al., 2012; Zangger et al., 2013).

The delta threshold cycle (Δct) values for each tested gene were obtained by calculating the difference between the ct value for the gene of interest and the geometric mean of the ct values of two housekeeping genes (*GAPDH* and *RPS18*). Data was centered and normalized by calculating the ΔΔct value. For each gene evaluated, we subtracted the geometric mean of the Δct values for the healthy control samples to the Δct for every sample, to obtain the ΔΔct values. The ΔΔct values were directly employed in all subsequent analyses and are provided in Table S2.

### Differential Gene Expression Analysis

To identify genes differentially expressed between healthy and lesioned skin and between *L. tropica* and *L. major* lesions, we employed the Comparative Marker Selection package from the GenePattern platform (v3.9.2, Broad Institute) (Gould et al., 2006). All differentially-expressed genes (DEGs) (*t* test, *P* < 0.05) between the two classes under analysis were extracted using the Extract Comparative Marker Selection package and are provided in Tables S3, S4.

### Hierarchical Clustering, Principal Component Analysis, and Correlation Matrices

The R Language and Environment for Statistical Computing (R) (v3.2.2) along with the RStudio interface (v0.99.892) were employed for PCA, Hierarchical Clustering, and plotting heatmaps and correlation matrixes. The *FactoMineR* and *factoextra* R packages were employed to perform PCA for genes and samples. The *hclust* R function was employed to cluster samples. The *heatmap.2* and *cormat* functions were employed to plot heatmaps and correlation matrices, respectively.

### Parasite and Mouse Macrophage Cell Line Culture

A *L. major* reference strain (MHOM/IL/81/Friedlin) was maintained in culture by weekly sub-passage 1X M199, 25 mM of HEPES, 1X Penicillin-Streptomycin, 1X glutamine, 0.0001% of biotin, 10 μg/mL of folic acid, 100 μM of adenosine, 5 μg/mL of hemin and 10% of heat inactivated FBS. The murine macrophage cell line J774 was acquired from ATCC and maintained in culture by regular sub-passage in RPMI culture media supplemented with FBS and antibiotics.

### Human Monocyte-Derived Macrophage (MDM) Differentiation

Peripheral blood mononuclear cells (PBMC) were purified from the peripheral blood using Ficoll-Paque (GE Healthcare) and density centrifugation. Monocytes were isolated by positive selection using CD14+ magnetic microbeads (Miltenyi) and differentiated into macrophages by culture in RPMI supplemented with 5% fetal calf serum (FCS; Gibco), 5% human serum (Sigma), penicillin-streptomycin (Gibco), and 25 ng/ml macrophage colony-stimulating factor (M-CSF; ImmunoTools).

### *In vitro* Infections and Quantitative PCR

Human MDMs and J774 macrophages were infected with *L. major* stationary-phase promastigotes at a parasite-to-cell ratio of 10-to-1, for 4 h, after which non-internalized parasites were removed through extensive washing in PBS. Cells were then treated with a TDO-specific inhibitor (680C91, Sigma Aldrich) at a concentration of 20 μM, previously determined as non-toxic for both parasite and cells, or DMSO (vehicle). At 48 h after incubation at, cells were lysed in 800 μL of TRIzol reagent (Thermo Fisher Scientific. RNA was purified and reverse transcription and quantitative PCR using SensiFAST cDNA synthesis kit (Bioline) followed by a qPCR (SensiFAST SYBR Hi-ROX kit, Bioline). For gene amplification, mixtures were composed of SensiFAST buffer (2X, Bioline) and 200 nM of forward and reverse primer. Assays were performed in 10 μl reactions volume with 15 ng of cDNA sample. Thermocycling settings consisted of one hold of 2 min at 95°C followed by a two-step temperature (95°C for 15 s and 60°C for 30 s) over 40 cycles in a CFX384 touch real time PCR detection system (Bio-rad). To calculate the relative intracellular parasite growth between DMSO-treated and TDO-inhibitor treated cells, we quantified the parasite-specific transcripts *KMP11* and *RRNA45*, using *GAPDH* and *RPS18* as host reference genes.

## Results

### Cutaneous Leishmaniasis Is Associated With the Induction of a Pro-Inflammatory Gene-Signature and the Downregulation of Genes Associated With Epidermal Integrity and Fatty Acid and Arginine Metabolism

To compare the transcriptional profile of human CL lesions with healthy skin, we performed a qPCR analysis on 19 biopsies recovered from patients residing or visiting endemic regions in Morocco and three healthy skin controls from non endemic Moroccan areas (Table 1). We evaluated the transcript levels of a total of 170 genes. These were categorized according to the biological processes they are involved in (Table S1). Among these, 71 genes are involved in the inflammatory/immune responses, including cytokines, chemokines, and immune cell surface markers. Interferon-stimulated genes (ISGs) were also included in the analysis (44 genes) as they have been implicated in the innate response to *Leishmania* infection *in vitro* (Favila et al., 2014), as well as in mice (Schleicher et al., 2018), but have not so far been examined with detail in cutaneous lesions from human patients. We further included 47 genes whose products fulfill metabolic roles that have a known impact on immune cell activation and function (Pearce and Pearce, 2013), as *Leishmania* has been described as capable of modulating the metabolic profile of the host cell (Moreira et al., 2015). Finally, we also included genes that ensure the maintenance of the skin barrier function (8 genes), as cutaneous leishmaniasis negatively impacts on epidermal integrity (Novais et al., 2015). Thus, the diversity of this group of selected genes should enable us to, first identify new genes that are differentially expressed between normal skin and lesions; second, to define a gene signature allowing the distinction of CL lesions caused by different species of *Leishmania* and, third, to identify genes whose expression in lesions is impacted by the parasite load.

**Table 1:** Demographic and clinical data from the subjects included in the cohort.

**Reference**	**Age/years**	**Gender**	Lesion evolution/months^*^	***Leishmania* species**
Healthy#1	32	M	–	–
Healthy#2	47	F	–	–
Healthy#3	53	F	–	–
CL#1 L02/12	57	F	2	*L. major*
CL#2 L07/12	26	M	9	*L. major*
CL#3 L45/12	56	F	2	*L. major*
CL#4 LC10/12	1	F	7	*L. major*
CL#5 LC11/12	28	M	3	*L. major*
CL#6 LC13/12	62	F	36	*L. major*
CL#7 LC14/13	65	F	9	*L. major*
CL#8 LC16/14	11	M	6	*L. major*
CL#9 LC20/14	26	M	7	*L. major*
CL#10 LC21/14	19	F	8	*L. major*
CL#11 L27/12	1	F	4	*L. tropica*
CL#12 L12/12	5	F	7	*L. tropica*
CL#13 LC13/13	32	F	6	*L. tropica*
CL#14 L29/12	6	M	7	*L. tropica*
CL#15 L32/12	85	F	2	*L. tropica*
CL#16 LC05/12	29	M	6	*L. tropica*
CL#17 LC06/12	62	F	6	*L. tropica*
CL#18 LC08/12	18	F	2	*L. tropica*
CL#19 LC12/12	6	F	6	*L. tropica*

* *Time span, in months, between the appearance of the lesions and the medical consultation*.

**Figure 1:**
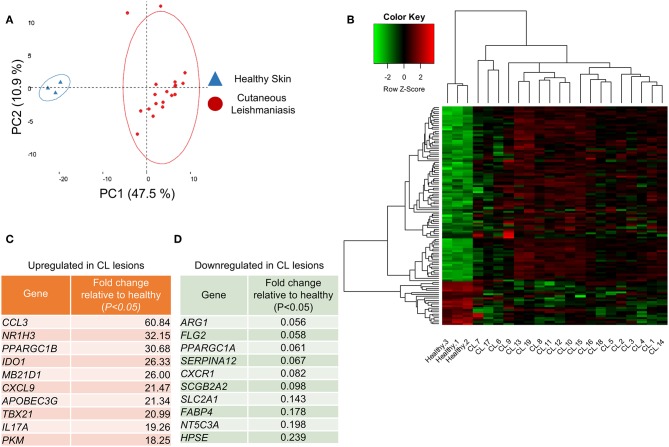
Healthy skin is transcriptionally distinct from cutaneous leishmaniasis lesions. **(A)** PCA analysis of the 170 analyzed genes from the 22 skin biopsies from either healthy skin (blue triangles) or CL patients (red dots). The two principal components (PC) are displayed on the axis along with the variance. Large circles denote the 95% confidence level. **(B)** Clustered heatmap of skin biopsies from CL patients according to their differential gene expression of healthy skin and CL lesions. **(C)** Table detailing the 10 most upregulated genes in CL lesions compared to healthy skin. **(D)** Table detailing the 10 most downregulated genes in CL lesions compared to healthy skin.

A principal component analysis (PCA) on the whole gene set resolved the samples into two groups, healthy controls and lesions, across the principal component-1, which accounted for 47.5% of the variation observed in the data (Figure 1A). Of the 170 evaluated genes, 111 were differentially expressed between normal and CL skin (Table S3, *P* < 0.05). Hierarchical clustering on differentially-expressed genes (Figure 1B, rows) showed that genes segregate into those that were significantly upregulated in CL skin, as compared to normal skin (89 genes, Table S3, *P* < 0.05), and those whose expression decreases in lesions (22 genes, Table S3, *P* < 0.05). Figures 1C,D show the most up- and down-regulated genes, respectively. The gene encoding the chemokine CCL3 was the most upregulated in lesions (Figure 1C), while *Arg1* expression decreased about 18 times in lesions, as compared to healthy skin and was the most down-regulated gene in our analysis (Figure 1D).

Among the significantly up-regulated genes were those associated with Th1 function (*IFNG, TNF, TBX21*) (Figure S1A), cytotoxicity (*PRF1, GZMB, GNLY)* (Figure S1B), or inflammatory chemokines (*CXCL9, CCL3, CCL5*) (Figure S1C). Similarly, genes encoding for inflammasome components (*NLRP3, CASP1, CASP4*) (Figure S1D) were significantly enriched in lesions, as compared to healthy skin, as well as genes encoding for Th17 cytokines (*IL17A, IL22*) (Figure S1E) and immune regulation (*IL10, TGFB1)* (Figure S1F). Finally, several Interferon-stimulated genes (ISGs) were also significantly upregulated in CL lesions (*TRIM56, TREX1, IFI44L*) (Figure S1G).

In contrast, genes associated with Th2 CD4 T cell function (*IL4, IL13*) remained unchanged in lesions (Figure S1H). Among the most downregulated genes were those involved in the epidermal barrier function (*SERPINA12, FLG2, HPSE*) and fatty acid biosynthesis (*ACACA, FABP4, FASN*) (Figure 1D and Figures S1I,J).

We further observed a significant upregulation of transcripts encoding for T cell surface markers (*CD4, CD8A, CD3G*) (Figure S2A), B cell markers (*MS4A1*) (Figure S2B) or neutrophils (*MPO*) (Figure S2C), but a significant decrease in the transcript levels of the blood monocyte marker *CD14* (Figure S2D).

Altogether, our data suggests that *Leishmania* induces the recruitment of immune cells to the site of cutaneous infection, triggering an immune response characterized by both pro-inflammatory and regulatory immune factors.

### Up-Regulation of a Cytotoxic/Inflammatory Signature in CL Lesions Leads to Compromised Expression of Genes Involved in Epidermal Integrity and Arginine Metabolism

**Figure 2:**
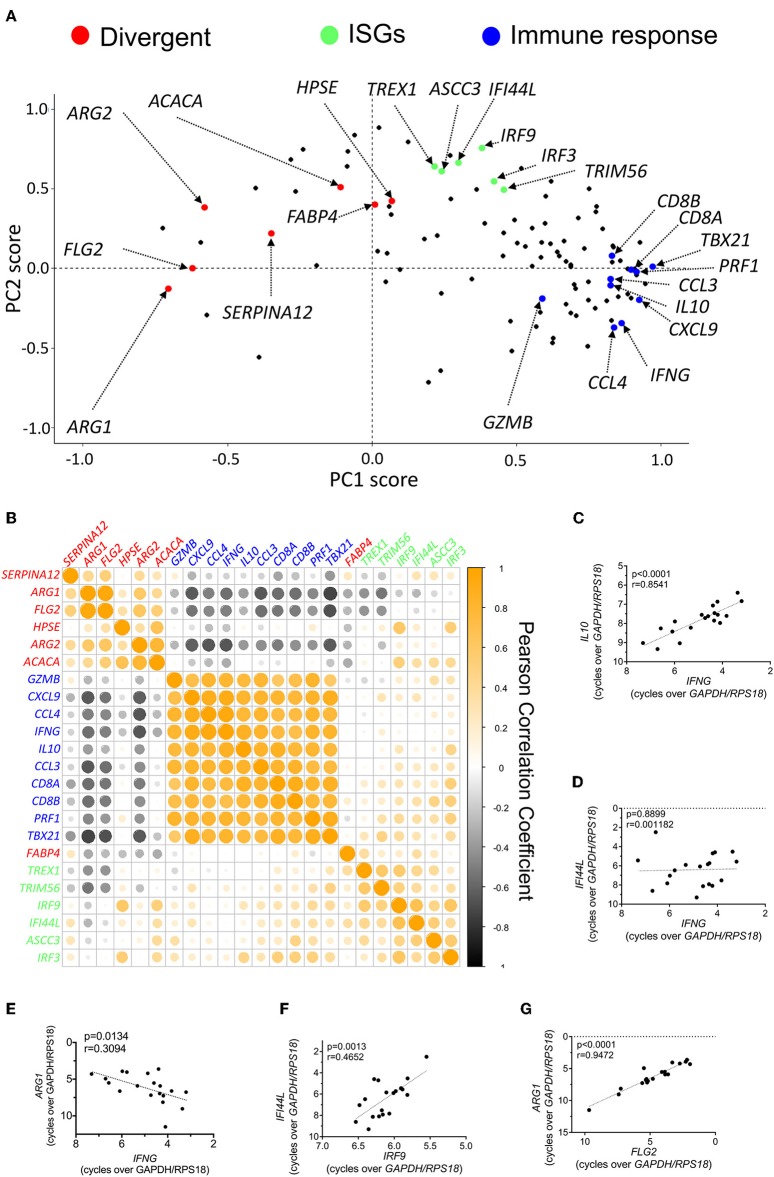
Induction of a pro-inflammatory signature in CL skin is associated with a dampened expression of genes involved in epidermal integrity and arginine and fatty acid metabolism. **(A)** Scores plot from PCA of skin biopsies from CL patients showing the PCA scores for each of the 111 differentially-expressed genes. Certain groups of genes are highlighted due to their comparable PCA scores and functional relationship. **(B)** Clustered heatmap of Pearson correlation coefficients of the expression levels for the genes highlighted in Figure 4A in infected skin biopsies. **(C–G)** Pearson correlation of the expression levels of selected pairs of genes from Figure 4B across infected biopsies; **(C)** *IL10* vs. *IFNG*; **(D)** *IFI44L* vs. *IFNG*; **(E)** *ARG1* vs. *IFNG*; **(F)** *IFI44L* vs. *IRF9*; **(G)** *ARG1* vs. *FLG2*.

To gain additional insight into the transcriptional signatures operating in CL, we performed a new PCA, now limited to the 111 differentially-expressed genes identified above, and to the 19 biopsies recovered from CL patients (Figure S3). The two principal components accounted for about 50% of the variation observed across the lesion samples (Figure S3). To identify the genes, or groups of genes that contribute mostly to such variation, we plotted the individual contributions of each gene to these two principal components (Figure 2A). This approach should allow the identification of groups of genes that are co-induced or co-repressed in individual samples. We observed that a group of genes involved in the immune response (including *CD8, TBX21, PERF1, GZMB, IFNG*, and chemokines such as *CXCL19, CCL3, CCL4*) are clustered together in the PCA scores plot (Figure 2A, blue dots). Similarly, several ISGs (including *IRF9, IRF3, TREX1, IFI44L*, and *TRIM56*), appear closely grouped (Figure 2A, green dots), while a third group of functionally-diverse genes appear segregated (“Divergent”) from both the “ISG” and “Immune Response” groups (Figure 2A, red dots). This latter group is comprised of genes whose expression decreases in lesions as compared with healthy skin, and play functions in epidermal integrity, and fatty acid and arginine metabolism, including, among others, *ARG2, ACACA*, or *FABP4* (Figure 2A, red dots).

Individual genes belonging to a given group should appear strongly co-induced or co-repressed in individual patients but present no association or correlate negatively with genes in other groups. To visualize this, we plotted the Pearson correlation coefficients for the genes selected for each of the three groups (Figure 2B). A complete Pearson correlation matrix for the whole differentially-expressed gene data set in patient biopsies is presented in Figure S4, and provides a comprehensive view of the simplified matrix presented in Figure 2B. Genes belonging to the “Immune Response” group, such as *IL10* and *IFNG*, present a strong positive correlation among them (Figures 2B,C), but bear no correlation with genes in the “ISGs” group (Figures 2B,D) or, instead, negatively correlate with most genes in the “Divergent” group, particularly those involved in epidermal integrity, or arginine metabolism (Figures 2B,E).

Similarly, individual genes in the “ISGs” group correlate positively with each other but have little association with genes in other groups (Figures 2B,D,F). These genes are also strongly induced in patient biopsies, as compared to healthy skin (Table S3). As the expression of ISGs is usually associated with viral infection, we assessed whether their induction in samples was due to the presence of the *Leishmania* RNA virus (LRV) (Zangger et al., 2013). Indeed, LRV2 was recently isolated from an Iranian strain of *L. major* (Hajjaran et al., 2016). However, LRV sequences were not detected in the skin biopsies of our cohort of patients (not shown).

These observations indicate that CL patients vary in their magnitude of induction of inflammatory genes and the potency of this program impacts on the mechanisms that maintain skin integrity.

### Lesions Caused by *L. major* or *L. tropica* Lead to Very Similar Transcriptional Profiles, the Latter Presenting an Aggravated Inflammatory Signature

**Figure 3:**
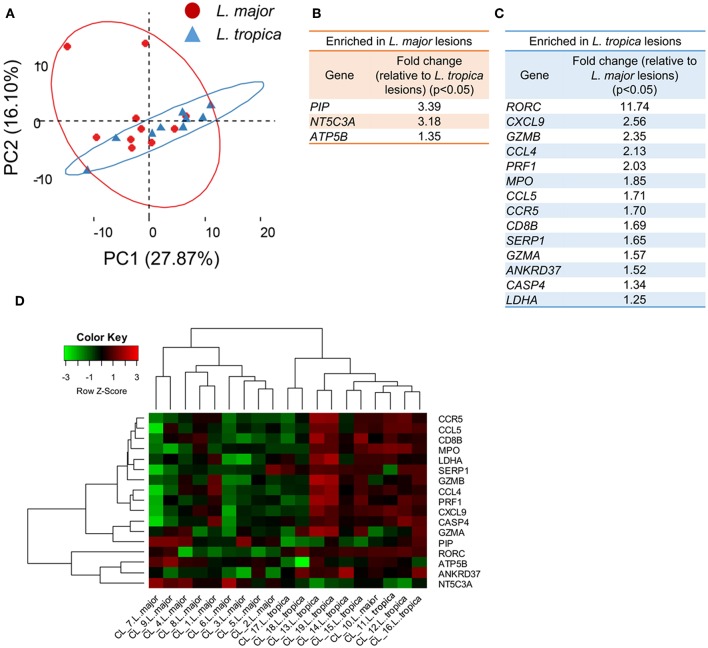
Skin biopsies from CL patients caused by *L. major* or *L. tropica* are transcriptionally very similar, the latter presenting an aggravated inflammatory/cytotoxic signature. **(A)** PCA analysis of the 170 analyzed genes from the 19 CL skin biopsies caused by infection with either *L. major* (red dots) or *L. tropica* (blue triangles). The two principal components (PC) are displayed on the axis along with the variance. Large circles denote the 95% confidence level. **(B)** Table detailing the genes significantly (*P* < 0.05) enriched in *L. major* biopsies as compared with *L. tropica*. **(C)** Table detailing the genes significantly (*P* < 0.05) enriched in *L. tropica* biopsies as compared with *L. major*. **(D)** Clustered heatmap of skin biopsies from CL patients according to their expression of the genes differentially expressed between *L. major* and *L. tropica* lesions.

Among the 19 patients in our cohort, 10 presented lesions caused by *L. major*, while the remaining were infected with *L. tropica* (Table 1). We asked whether the variability in the transcriptional profiles observed across patients could be related to distinct transcriptional responses induced by the two species. A PCA on the whole gene data set and limited to the 19 biopsies from patients could not resolve the samples into lesions caused by either species (Figure 3A), providing an indication that the transcriptional profile induced by both *Leishmania* species are very similar. We then compared the transcriptional profile of the 10 lesions caused by *L. major* with the nine samples presenting *L. tropica* lesions to extract the genes that are differentially-expressed between lesions caused by the two species (*P* < 0.05, Table S4). Only three genes (*PIP, NT5C3A*, and *ATP5B*), that are functionally-unrelated, were significantly upregulated in *L. major* lesions as compared to CL caused by *L. tropica* (*P* < 0.05, Figure 3B, Figure S5A, and Table S4). Fourteen genes were significantly upregulated in *L. tropica* lesions, as compared to *L. majo*r (Figure 3C). Among these, many were involved in the CD8 T cell immune response (*GZMA, GZMB, PRF1*, and *CD8B*), and also included genes encoding for inflammatory chemokines such as *CCL5, CCL4* and its ligand *CCR5*, as well *CXCL19*, which plays a critical role in the recruitment of CD8 T cells (Hugues et al., 2007) (Figure 3C, Figure S5B, and Table S4). We also observed higher levels of *RORC* expression in *L. tropica* lesions, a transcriptional factor reported to be expressed by CD8 T cells of psoriatic skin lesions (Ortega et al., 2009). While most of these transcripts are also upregulated in *L. major* lesions as compared to normal skin (Figure S5B, Table S3), our analysis revealed that they are further enriched in CL caused by *L. tropica*.

We next asked whether the 17 differentially-expressed genes could constitute a signature able to distinguish and group together lesions caused by each of the species. For that, we clustered the samples from the 19 infected patients based on their expression levels of these 17 genes (Figure 3D). This approach yielded a relatively good separation of the samples according to the parasite species, since only one of the *L. major*-caused lesions segregated into the *L. tropica* group (CL10) (Figure 3D). We cannot exclude that additional host or environmental factors in this particular patient may contribute to blur the signatures induced by the two species.

We conclude that, within our gene dataset, both *Leishmania* species induce very similar gene signatures in the infected skin. Nevertheless, our results indicate that CL lesions caused by *L. tropica* bear an aggravated inflammatory profile as compared to *L. major* lesions that appears to result from increased CD8 T cell responses at the site of infection.

### The Transcript Levels of the Tryptophan-Metabolizing Enzyme TDO Negatively Correlate With the Parasite Load in Lesions, and Inhibition of TDO Activity Promotes Intracellular *L. major* Growth in *in vitro*-Infected Macrophages

**Figure 4:**
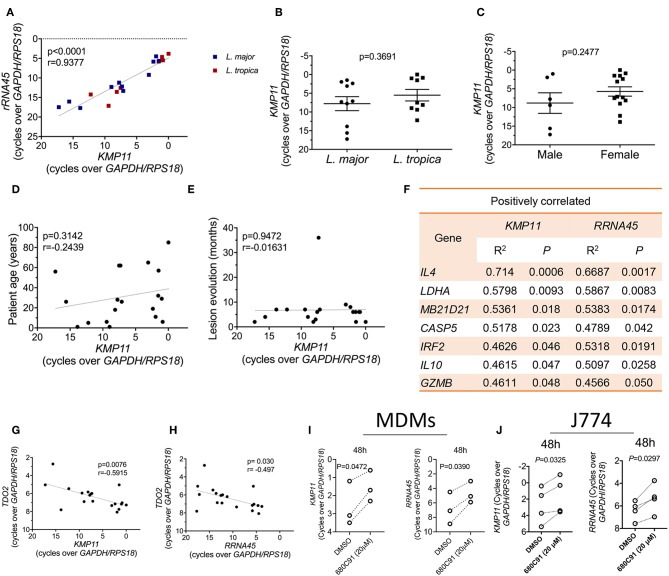
TDO gene expression is inversely correlated with the parasite load in lesions and inhibition of TDO boosts the parasite load in *L. major*-infected macrophages. **(A)** Correlation between the transcript levels of the *Leishmania* genes KMP11 and RRNA45 in CL biopsies from patients infected with *L. major* or *L. tropica* (Pearson correlation coefficient). **(B)** Comparison of the level of KMP11 expression between *L. major* and *L. tropica* biopsies (unpaired *T*-test). **(C)** Comparison of the level of *KMP11* expression between biopsies from male and female patients (unpaired *T*-test). **(D)** Correlation between the level of *KMP11* expression and the age of CL patients (Pearson correlation coefficient). **(E)** Correlation between the level of expression of *KMP11* and the time in months after lesion appearance (Pearson correlation coefficient). **(F)** Table of genes whose expression is significantly (*P* < 0.05) positively correlated with the expression levels of both *KMP11* and *RRNA45*. **(G)** Correlation between the levels of mRNA expression of *KMP11* and *TDO2* (Pearson correlation coefficient). **(H)** (Correlation between the levels of mRNA expression of *RRNA45* and *TDO2* (Pearson correlation coefficient). **(I,J)** MDMs **(I)** or J774 cells **(J)** were infected with *L. major* stationary phase promastigotes and subsequently treated with 20 μM of the TDO specific inhibitor, 680C91 or vehicle (DMSO). Cells were lysed for RNA extraction at 48 h after infection. The transcript levels of the *Leishmania* genes *KMP11* (left panels) or *RRNA45* (right panels) were quantified by qPCR and normalized by the expression of the host housekeeping genes (*GAPDH* and *RPS18*) (paired *T*-test). **(A–E,G,H)** Each dot represents an individual CL skin biopsy. **(I)** Each paired dot represents an independent blood donor (*n* = 3). **(J)** Each paired dot represents an independent experiment (*n* = 4).

Recent data suggests that the parasite load in lesions impacts the type and magnitude of the immune responses induced during CL caused by *L. braziliensis* (Christensen et al., 2016). To evaluate whether a similar influence is present in lesions caused by *L. major* and *L. tropica*, we quantified the transcript levels of two parasite genes in the lesions, *KMP11* and *RRNA45*. These two genes are conserved across the *Leishmania* genus and stably expressed during the parasite's life cycle (Ouakad et al., 2007). They have also been validated as endogenous reference genes for gene expression studies in *Leishmania* (Moreira et al., 2012; Zangger et al., 2013), and thus should provide a precise quantification of the relative parasite load in samples. We observed that the transcript levels of these two parasite genes are strongly correlated with each other in skin biopsies, as expected (Figure 4A). At the limit of detection of our assay (CT ≤ 40), we could not amplify these transcripts from healthy skin samples (not shown). The variation in the parasite transcripts across the patients' samples wasn't due to the infecting species (Figures 4A,B), the patient's gender (Figure 4C), the patient's age (Figure 4D), or the time after lesion appearance (Figure 4E). We thus sought to identify host genes whose expression is either positively or negatively correlated with the parasite load. For that, we plotted the Pearson correlations between the expression levels of either *KMP11* or *RRNA45* and each of the host genes in our entire dataset. The expression of a total of seven genes were significantly positively correlated with the mRNA levels of both *KMP11* and *RRNA45* (Figure 4F). These include well-known susceptibility factors for CL, such as *IL4* or *IL10*. We also found that factors usually implicated in antiviral defense such as cGAS (*MB21D1)* and *IRF2* appear upregulated in lesions with higher parasite transcripts (Figure 4F).

Strikingly, among our entire dataset, a single gene, *TDO2*, which encodes for the tryptophan-catabolizing enzyme tryptophan 2,3-dioxygenase (TDO), negatively correlates with the expression levels of both *KMP11* and *RRNA45* (Figures 4G,H).

The negative correlation observed between the transcript levels of TDO and the parasite load in lesions, suggests that this enzyme may act as a restriction factor for cutaneous *Leishmania*. To test this hypothesis, we infected primary human monocyte-derived macrophages (MDMs) (Figure 4I) or the murine macrophage cell line J774 (Figure 4J) with *L. major* stationary phase promastigotes. After 4 h of infection, cells were treated with a specific TDO inhibitor, 680C91. We observed a significant increase in the levels of parasite transcripts at 48 h after treatment with 20 μM of 680C91, in both cell types, as measured by the expression of *KMP11* (Figures 4I,J, left panels). A similar result was observed when using *RRNA45* to quantify the parasite load (Figures 4I,J, right panels). We thus conclude that TDO restricts the intracellular growth of *Leishmania major* and its expression levels correlate negatively with the parasite transcripts in cutaneous biopsies.

## Discussion

The clinical presentation of CL results from a complex interplay of multiple factors, including the host's genetic background, its immunological status, and the parasite species (Scott and Novais, 2016). By exploring the variation in gene expression across lesion samples, we found that the induction of genes associated with immune responses was negatively correlated with the levels of transcripts encoding for genes ensuring epidermal integrity and fatty acid metabolism. This immune response group of genes was particularly enriched in genes involved the effector cytotoxic CD8 T cell function, including *CD8A, CD8B, PRF1* (perforin-1), and *GZMB* (granzyme B), as well as *TBX21* (T-bet). This group further comprised *CCL5* (Rantes) and *CCL4* (MIP-1β), which attract effector CD8 T cells to inflamed tissues (Franciszkiewicz et al., 2012). Our observations thus suggest that the magnitude of the CD8 cytotoxic response has a direct impact on the integrity of the skin and lesion pathology. Our data is in agreement with observations made on *L. braziliensis* lesions, where a similar relationship between the induction of a cytotoxic program and loss of epidermal integrity seems to operate (Novais et al., 2015, 2017).

We further observed the induction of a group of interferon-stimulated genes (ISGs) in CL biopsies, including transcripts encoding for IRF9, IRF3, IFI44L, and TRIM56. Interestingly, the induction of this type I IFN signature was independent of the main pro-inflammatory module and did not show any association with the expression of genes maintaining epidermal integrity or involved in fatty acid or arginine metabolism. Thus, our data suggests that the ISG signature does not directly contribute to the immunopathogenesis of CL, in contrast with what is observed in metastatic mucocutaneous leishmaniasis (MCL) (Rossi et al., 2017). As ISGs are generally induced following viral infection, we reasoned that the presence of LRV could explain the induction of these genes in a subset of the samples. We couldn't however detect LRV sequences in any of the biopsies of the cohort. Also, the induction of the ISG signature was independent of the parasite species and the parasite load. Thus, the factors that lead to the upregulation of these ISGs in a subset of CL lesions remain unidentified. It is possible that exogenous viral infections, known to be transmitted alongside *Leishmania* by the sand-fly vector, may trigger the detectable ISG response during CL (Rossi et al., 2017).

We further found a significant upregulation of genes encoding for inflammasome components in CL biopsies, including *NLRP3, CASP1*, and *CASP4*. Activation of the inflammasome at the site of *Leishmania* inoculation depends on co-transmission of microbes from the sand-fly gut, leading to IL-1β production and neutrophil recruitment (Dey et al., 2018), thus supporting observation of elevated *IL1B* levels as well as the neutrophil marker *MPO* in our cohort of biopsies.

A major novelty in our study is the direct comparison of the transcriptional profile of CL lesions caused by infection with two distinct *Leishmania* species. As both types of lesions were recovered from the same endemic areas, this analysis should provide an unbiased approach to assess the impact of the infecting species on the transcriptional profile of CL. Our data indicates that the transcriptional profiles of CL caused by *L. major* or *L. tropica* are very similar. Nevertheless, we found that lesions caused by *L. tropica* present an aggravated inflammatory profile with increased expression of genes associated with the CD8 T cell cytotoxic function and the inflammatory response. Conversely, we found that the expression of the gene encoding pyrimidine 5′-nucleotidase (NT5C3A) was significantly enriched in *L. major* lesions, as compared with *L. tropica*. NT5C3A suppresses cytokine production through inhibition of the NF-κB pathway (Al-Haj and Khabar, 2018) and may represent a negative feedback regulator of inflammatory cytokine signaling in *L. major*-caused CL, as compared with *L. tropica* lesions.

Clinically, lesions caused by *L. tropica* tend to be dryer and take longer to heal as compared to those caused by *L. major* (Masoudzadeh et al., 2017). This supports the idea that an aggravated inflammatory/ cytotoxic profile in *L. tropica* lesions prevents or delays the action of the mechanisms of skin regeneration. Future studies, employing larger gene sets, should aim to pinpoint whether the distinct clinical pictures observed in lesions caused by *L. major* and *L. tropica* conceal distinct transcriptional signatures. These studies can potentially be extended to additional parasite species that co-exist in the same endemic regions.

The impact of the parasite load on the transcriptional profile of CL lesions has been explored in a few studies (Kumar et al., 2009; Christensen et al., 2016; Pereira et al., 2017), but so far such knowledge has not been translated into the identification of genes that restrict parasite growth. Our approach of quantifying parasite transcripts as a proxy for the parasite load is validated by the finding that *IL4* was the host transcript that more significantly correlated with parasite transcripts in a positive manner. This confirms a previous study that uncovered a correlation between the levels of *IL4* expression and the parasite load in *L. tropica* lesions (Kumar et al., 2009). It is interesting to note that, in our study, the expression levels of *IL4* are not significantly changed in CL lesions, as compared with healthy skin, which confirms the notion that, in humans, CL does not develop because of the abnormal expansion of Th2 cells. This suggests that the variability in the levels of *IL4* expression across individuals before infection is a major factor in determining the parasite load after infection. In this sense, it is interesting to note that polymorphisms at the level of the *IL4* promoter are associated with increased risk of developing CL (Kamali-Sarvestani et al., 2006). Additionally, CL patients with a concomitant helminth infection, known to drive a polarized Th2 response characterized by high IL-4 levels, bear lesions that take longer to heal as compared with helminth-free patients (O'Neal et al., 2007).

The transcript levels of the tryphophan-metabolizing enzyme TDO were negatively correlated with the parasite load in lesions. Furthermore, inhibition of TDO promoted an increase in parasite transcripts in human and mouse macrophages infected *in vitro* with *L. major*. TDO catalyzes the first, and rate-limiting, step in the biosynthesis of NAD+, by converting tryptophan to N-formylkynurenine (Platten et al., 2012). Its expression is particularly enriched in the liver, where it regulates the systemic levels of tryptophan, but other tissues such as the brain, lungs, or skin also appear to express low levels of the enzyme (van Baren and Van den Eynde, 2015). Unlike indoleamine 2,3-dioxygenase-1 (IDO), the other major enzyme involved in the degradation of tryptophan, TDO is not induced by inflammatory stimuli or cytokines (Lanz et al., 2017). Unlike IDO, the role of TDO during the immune response remains mostly unexplored. In our study, while *IDO1* was among the genes most highly induced in lesions as compared to healthy skin, the levels of *TDO2* remained unchanged. However, the abundance of *IDO1* transcripts had no correlation with the parasite load in lesions, while *TDO2* correlated negatively with the levels of parasite transcripts. *Leishmania* is auxotrophic for tryptophan (Nayak et al., 2018), which suggests that TDO may repress parasite growth by depleting intracellular tryptophan levels. However, within the inflammatory environment that characterizes CL and the consequent strong induction of IDO, the role of TDO in regulating tryptophan levels is probably negligible. As such, TDO may restrict cutaneous *Leishmania* through a mechanism not related to the depletion of tryptophan. Alternatively, the subcellular distribution of TDO may be distinct from IDO and allow it to selectively deplete tryptophan in the vicinity of parasite compartments. However, little is known about the subcellular localization of TDO. It is interesting to note that the growth of intracellular pathogens is differentially impacted by the inhibition of tryptophan degradation. For instance, the administration of an IDO inhibitor to *Leishmania major*-infected mice led to a better control of the infection and decreased the parasite load. In contrast, inhibition of IDO in *Toxoplasma gondii*-infected mice led to uncontrolled parasite growth, and accelerated mortality (Divanovic et al., 2012). Thus, the capacity of tryptophan-depleting enzymes to control intracellular parasites is pathogen-specific, likely depending on factors such as the replication kinetics of the parasite, or the localization of its intracellular niche, with TDO apparently being better suited to restrict *Leishmania* growth. Finally, given the strong expression of TDO in the liver, it is of the utmost interest to assess the impact of TDO inhibition on the growth of liver-targeting *Leishmania* species, such as *L. donovani*.

The relationship between the pathology of CL and the parasite load in the lesion is complex. In general, diffuse manifestations of cutaneous leishmaniasis (DCL), caused for instance by *L. aethiopica* are characterized by large numbers of parasites in lesions that spread over vast areas of the skin. These patients exhibit severe T cell anergy against parasite antigen as well as CD8 T cell exhaustion (Hernandez-Ruiz et al., 2010), that appear to be promoted by elevated levels of regulatory cytokines, such as IL10 (Scott and Novais, 2016). On the opposite side of the spectrum, lesions presenting an aggravated inflammatory profile, associated with extensive tissue destruction and immunopathology, such as MCL caused by *L. braziliensis*, tend to bear very low numbers of parasites (Scott and Novais, 2016). These lesions are characterized by extensive infiltration of CD4 T cells with a polarized Th1 profile, as well as cytotoxic CD8 T lymphocytes (Faria et al., 2005). Both of these extremes are characterized by severe pathology. In one case, due to parasite proliferation, and the other due to an excessive immune response (Scott and Novais, 2016). In patients with localized cutaneous leishmaniasis a compromise is attained in which the parasite load is kept in check by an immune response whose magnitude is regulated by the action of regulatory cytokines such as IL-10 or TGF-β (Scott and Novais, 2016). Indeed, we observed a co-induction of these immunoregulatory factors along with the pro-inflammatory mediators, suggesting that they act as brakes to moderate an excessive immune response. However, even localized CL lesions take long to heal, often leaving disfiguring scars that are a source of social stigma for individuals. In this context, novel therapeutic interventions, such as inducing the activation of TDO, that could adjuvate conventional anti-*Leishmania* drugs may allow for faster healing of the lesions in the context of a milder inflammatory response. This has a clear advantage over classical immunotherapeutic approaches, such as the neutralization of regulatory cytokines or the inhibition of immune checkpoints, that often result in exacerbated immune responses and concomitant pathology.

## Data Availability Statement

All datasets generated for this study are included in the manuscript/Supplementary Files.

## Ethics Statement

This work was conducted according to the principles specified in the Declaration of Helsinki and under the local ethical guidelines of the Ethics Committee for Biomedical Research (Faculty of Medicine and Pharmacy, Hassan II University of Casablanca, Morocco) that approved this research. The team explained to the patients the objectives of the survey, and why it needed a cutaneous biopsy, which is used routinely at the Department of Dermatology (University Hospital Ibn Rochd, Casablanca) for the parasitological confirmation of cutaneous leishmaniasis diagnosis before any treatment prescription. The dermatologist asked for the patients consent (for adults) or from the parents for children. The sampling was done only if the patients or their tutors gave their oral consent. At the time of biopsy sampling (between 2012 and 2014), oral consent was the sole requirement imposed by the Ethics Committee to allow for patient tissue sampling for research purposes and thus written consent was not obtained. Oral consent was also obtained from the healthy skin donors. Finally, the team guaranteed the confidentiality of their personal and clinical data and that the results would be processed anonymously.

## Author Contributions

VR, MR, KA, and JE planned research and coordinated the study. VR, SA, HM, and TM performed the experiments. SC provided the clinical samples. VR analyzed the data. VR and JE wrote the manuscript with contributions from MR and KA. All authors read and approved the submitted version of the manuscript.

### Conflict of Interest

The authors declare that the research was conducted in the absence of any commercial or financial relationships that could be construed as a potential conflict of interest.
