# Supplementary material for: Transcriptional Analysis of Human Skin Lesions Identifies Tryptophan-2,3-Deoxygenase as a Restriction Factor for Cutaneous Leishmania
Source: Front Cell Infect Microbiol. 2019 Oct 4;9:338. doi: 10.3389/fcimb.2019.00338 (PMC6788307; doi:10.3389/fcimb.2019.00338)
Supplement: Supplementary file 5 [file Data_Sheet_1.PDF]

Supplementary Figure Legends

Figure S1. The transcriptional signature of the CL lesion is characterized by the induction of pro-inflammatory transcripts, and the downregulation of genes linked with epidermal integrity and fatty acid metabolism.

A-I- Comparison of the level of expression for the indicated genes between biopsies from healthy skin or cutaneous leishmaniasis lesions. A- Th1 markers (*IFNG*, *TNF*, *TBX21*); B- Cytotoxicity markers (*PRF1, GZMB, GNLY*); C- chemokines (*CXCL9, CCL3, CCL5*); D- inflammasome components (*NLRP3, CASP1, CASP4*); E- Th17 cytokines (*IL17, IL22*); F- Regulatory cytokines (*IL10, TGFB1*); G-Th2 cytokines (*IL4, IL13*); H- Interferon-Stimulated Genes (*TRIM56*, *TREX1*, *IFI44L*); I-factors involved in epidermal integrity (*SERPINA12, FLG2, HPSE*); J- factors involved in fatty acid metabolism (*FABP4, ACACA, FASN*). Each dot represents an individual biopsy. Unpaired T test.

Figure S2. Increased transcript expression of diverse immune cell markers characterizes the cutaneous leishmaniasis lesion.

A-D- Comparison of the level of expression for the indicated genes between biopsies from healthy skin or cutaneous leishmaniasis lesions. A- T cell markers (*CD3G, CD4, CD8A*); B- B cell marker (*MS4A1*); C- neutrophil marker (*MPO*); D- monocyte marker (*CD14*). Each dot represents an individual biopsy. Unpaired T test.

Figure S3. PCA analysis of the 111 differentially-expressed genes from the 19 skin biopsies from CL patients. The 2 principal components (PC) are displayed on the axis along with the variance.

Figure S4. Clustered heatmap of Pearson correlation coefficients of the expression levels for the 111 differentially-expressed genes.

Figure S5.

A- Comparison of the expression levels of *NT5C3A, PIP* and *ATP5B* expression between healthy, *L. major* and *L. tropica* biopsies (unpaired T test between *L. major* and *L. tropica* lesions).

B- Comparison of the expression levels of *RORC, CXCL9, GZMB, CCL4, PRF1, MPO, CCL5, CCR5, CD8B, CASP4, SERP1, GZMA, ANKRD37* and *LDHA* expression between healthy, *L. major* and *L. tropica* biopsies (unpaired T test between *L. major* and *L. tropica* lesions. Each dot represents an individual CL skin biopsy.
