# Supplementary material for: Transcriptional Analysis of Human Skin Lesions Identifies Tryptophan-2,3-Deoxygenase as a Restriction Factor for Cutaneous Leishmania
Source: Front Cell Infect Microbiol. 2019 Oct 4;9:338. doi: 10.3389/fcimb.2019.00338 (PMC6788307; doi:10.3389/fcimb.2019.00338)
Supplement: Supplementary file 6 [file Data_Sheet_2.PDF]

Supplementary Table Legends

Table S1. List of primers employed in the study and their sequences.

Table S2. Values for the ΔΔct for all the transcripts amplified from the skin biopsies

Table S3. List of all the genes differentially expressed between normal and lesional skin ranked by their enrichment in lesion skin.

Table S4. List of all the genes differentially expressed between *L. major* and *L. tropica* lesions ranked by their enrichment *L. major* lesions
