# Supplementary figures and images for: Transcriptional Analysis of Human Skin Lesions Identifies Tryptophan-2,3-Deoxygenase as a Restriction Factor for Cutaneous Leishmania
Source: Front Cell Infect Microbiol. 2019 Oct 4;9:338. doi: 10.3389/fcimb.2019.00338 (PMC6788307; doi:10.3389/fcimb.2019.00338)

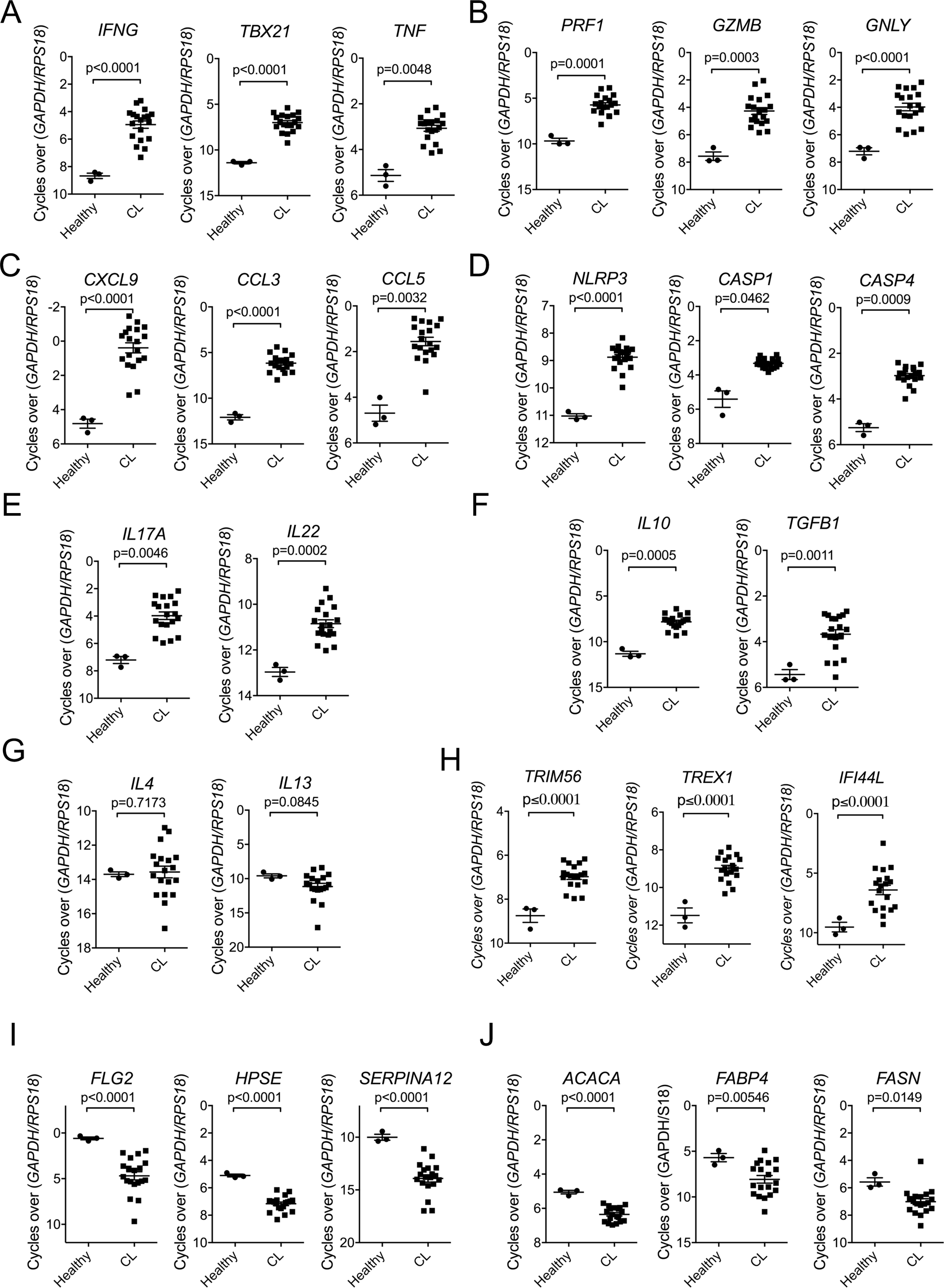

Supplement: Supplementary file 7 [file Image_1.TIF]

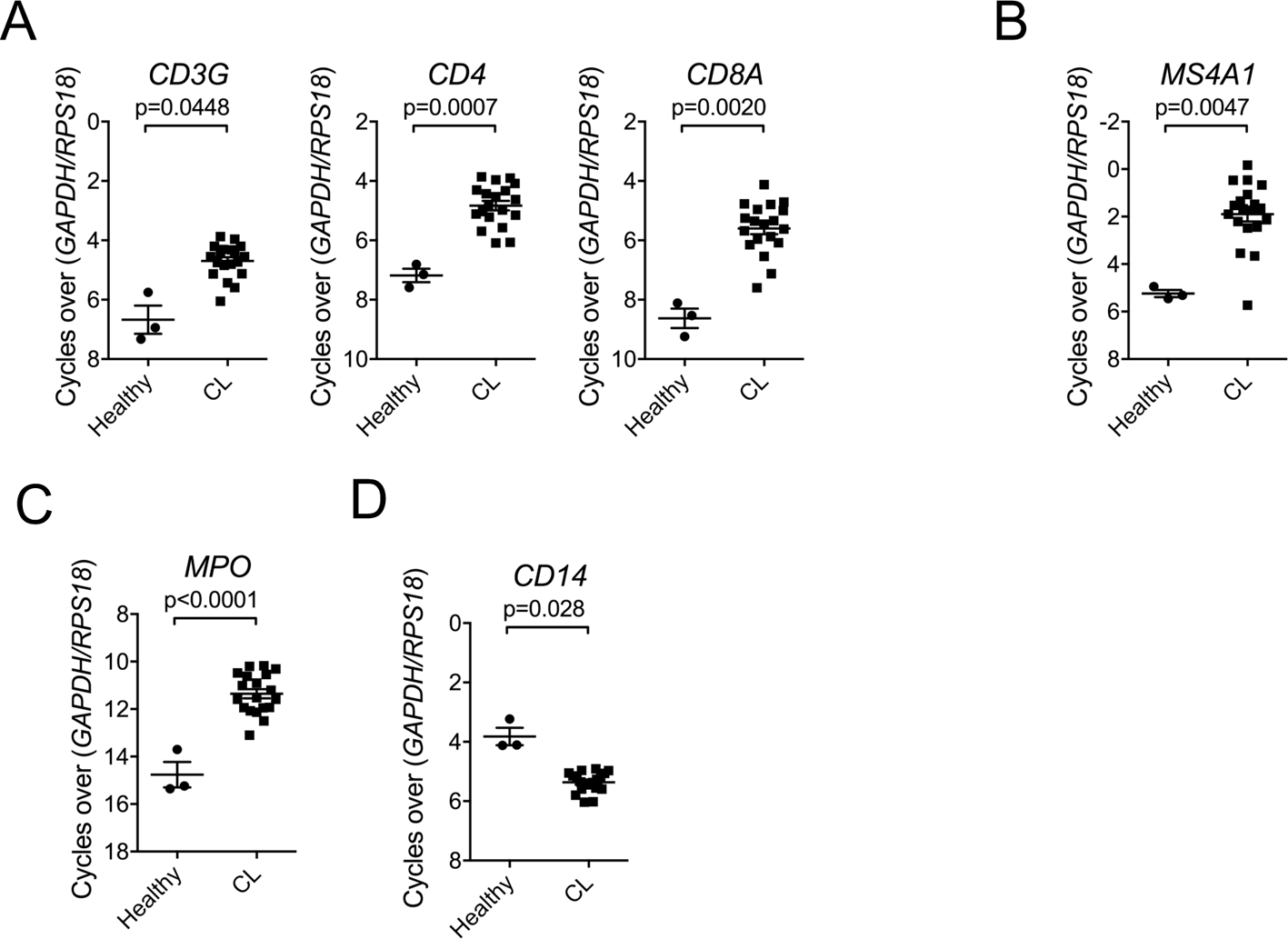

Supplement: Supplementary file 8 [file Image_2.TIF]

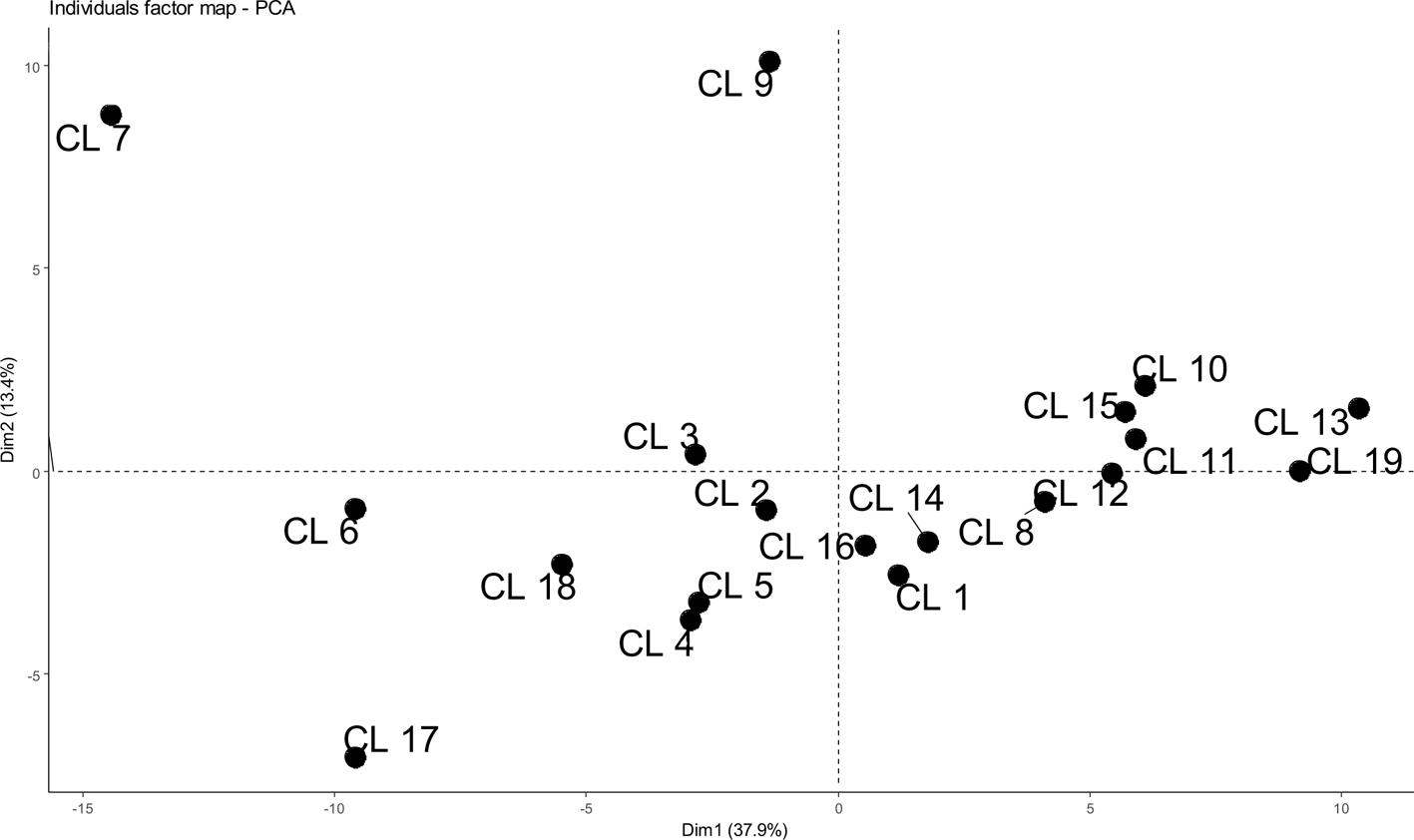

Supplement: Supplementary file 9 [file Image_3.TIF]

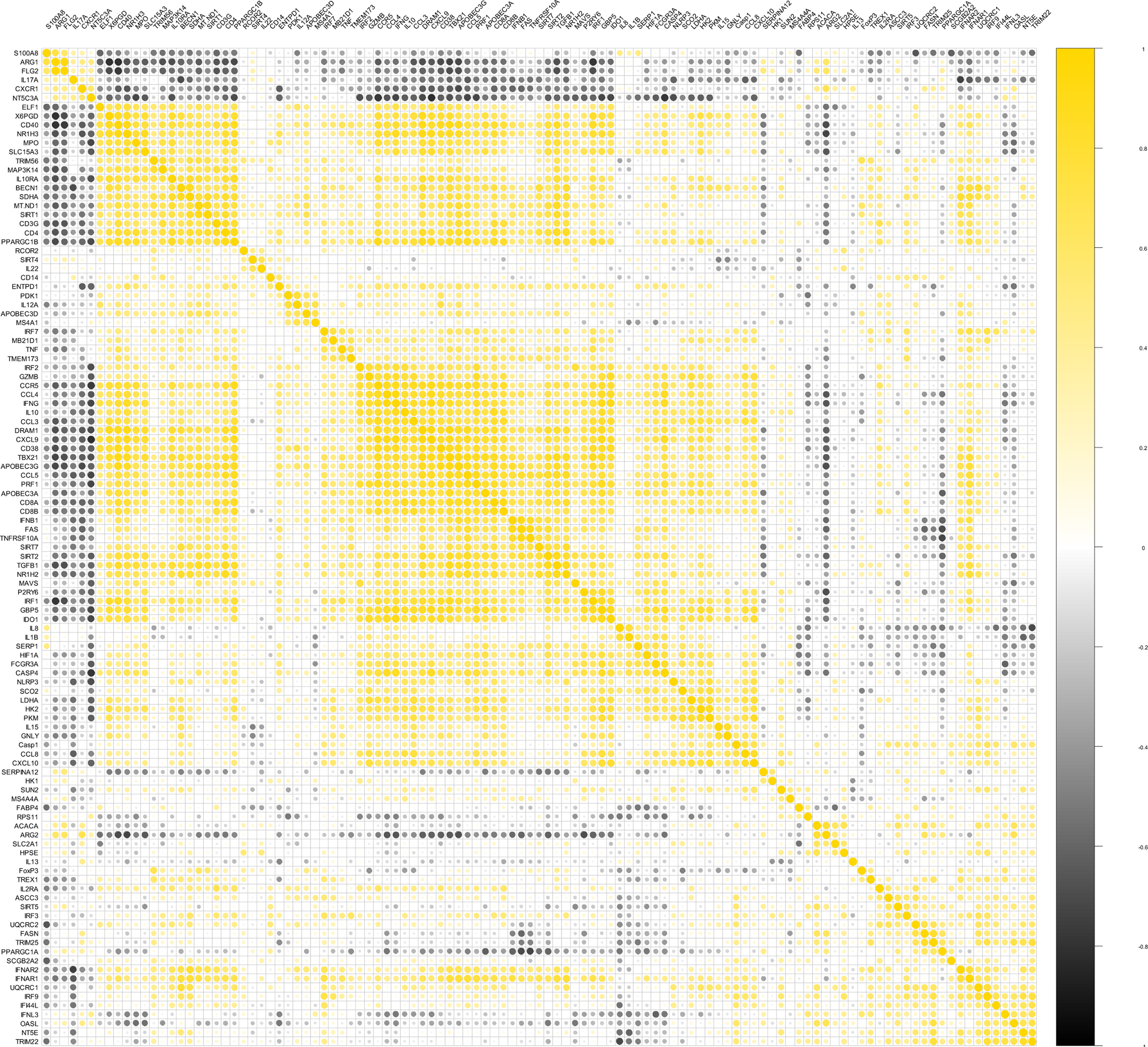

Supplement: Supplementary file 10 [file Image_4.TIF]

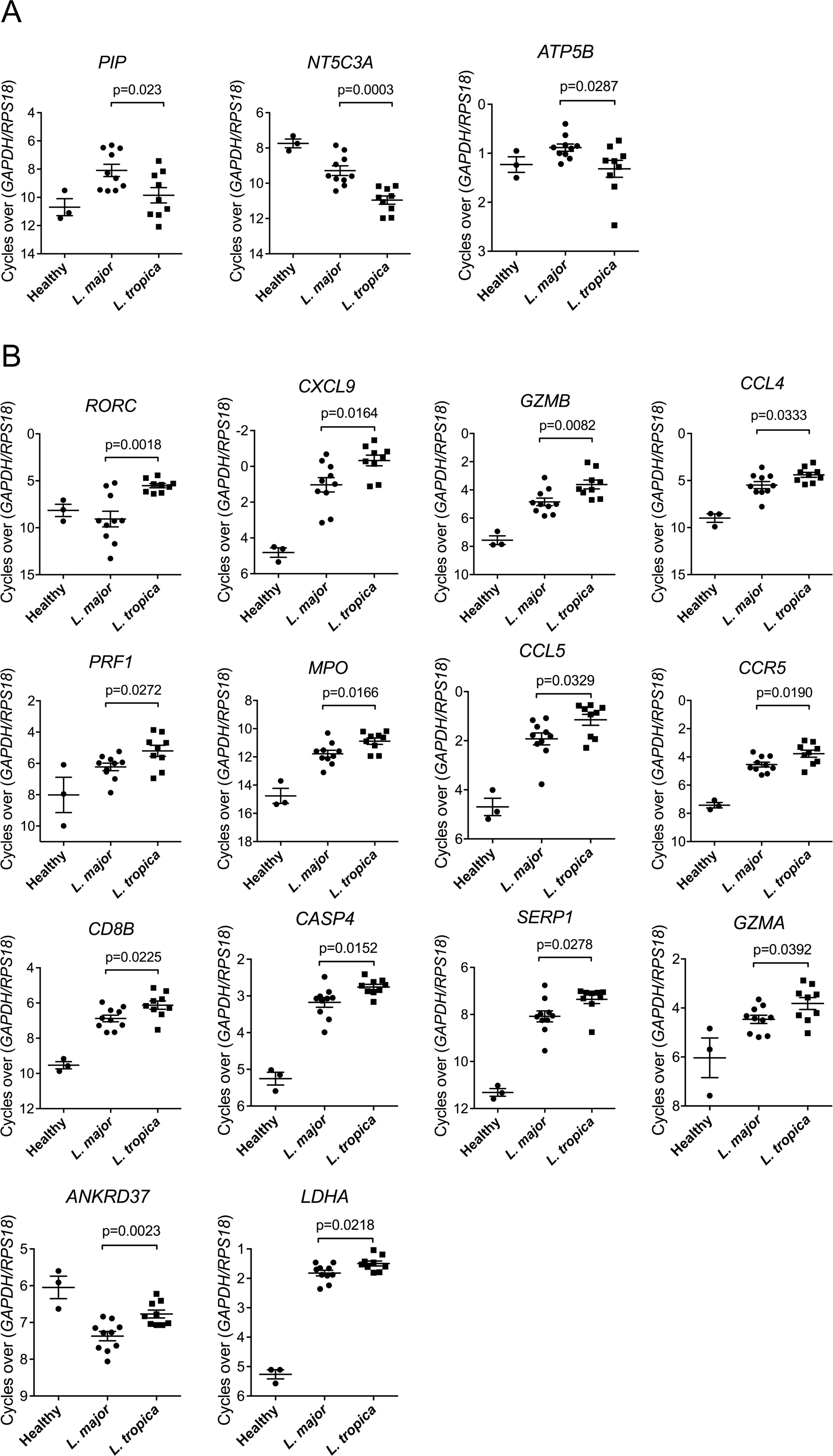

Supplement: Supplementary file 11 [file Image_5.TIF]
